# Discontinuous and Continuous Indoor Air Quality Monitoring in Homes with Fireplaces or Wood Stoves as Heating System

**DOI:** 10.3390/ijerph13010078

**Published:** 2015-12-24

**Authors:** Gianluigi de Gennaro, Paolo Rosario Dambruoso, Alessia Di Gilio, Valerio Di Palma, Annalisa Marzocca, Maria Tutino

**Affiliations:** 1 Department of Biology, University of Bari Aldo Moro—Via Orabona 4, Bari 70126, Italy; v.d.palma@tue.nl; 2 Apulia Regional Agency for Environmental Prevention and Protection—Corso Trieste 27, Bari 70126, Italy; p.dambruoso@arpa.puglia.it (P.R.D.); a.digilio@arpa.puglia.it (A.D.G.); a.marzocca@arpa.puglia.it (A.M.); m.tutino@arpa.puglia.it (M.T.)

**Keywords:** fireplace, stove, wood combustion, indoor air quality, ultrafine particles

## Abstract

Around 50% of the world’s population, particularly in developing countries, uses biomass as one of the most common fuels. Biomass combustion releases a considerable amount of various incomplete combustion products, including particulate matter (PM) and polycyclic aromatic hydrocarbons (PAHs). The paper presents the results of Indoor Air Quality (IAQ) measurements in six houses equipped with wood burning stoves or fireplaces as heating systems. The houses were monitored for 48-h periods in order to collect PM_10_ samples and measure PAH concentrations. The average, the maximum and the lowest values of the 12-h PM_10_ concentration were 68.6 μg/m^3^, 350.7 μg/m^3^ and 16.8 μg/m^3^ respectively. The average benzo[a]pyrene 12-h concentration was 9.4 ng/m^3^, while the maximum and the minimum values were 24.0 ng/m^3^ and 1.5 ng/m^3^, respectively. Continuous monitoring of PM_10_, PAHs, Ultra Fine Particle (UFP) and Total Volatile Organic Compounds (TVOC) was performed in order to study the progress of pollution phenomena due to biomass burning, their trends and contributions to IAQ. The results show a great heterogeneity of impacts on IAQ in terms of magnitude and behavior of the considered pollutants’ concentrations. This variability is determined by not only different combustion technologies or biomass quality, but overall by different ignition mode, feeding and flame management, which can also be different for the same house. Moreover, room dimensions and ventilation were significant factors for pollution dispersion. The increase of PM_10_, UFP and PAH concentrations, during lighting, was always detected and relevant. Continuous monitoring allowed singling out contributions of other domestic sources of considered pollutants such as cooking and cigarettes. Cooking contribution produced an impact on IAQ in same cases higher than that of the biomass heating system.

## 1. Introduction

Indoor Air Quality requires attention as it relates to the health and comfort of people that spend most of their time indoors [1,2,3]. Heating, cooking, smoking, cleaning as well as furnishings or building materials are important indoor sources of gaseous pollutants and particles [4,5,6,7,8]. The impact of these sources is linked to the amount and hazard of the emitted pollutants [9,10]. Moreover, several factors such as occupant’s behavior, microclimatic and ventilation condition and outdoor intrusion can influence indoor pollution levels [11]. Great interest is paid to particulate matter (PM) in relation to its concentration, chemical composition and the duration of exposure [8]. A large number of indoor particle sources were identified and investigated by many studies. Among these, resuspension of particles by human activities and pet movements, dusting, vacuuming and showering contributes to the coarse mode of indoor particles [12,13,14]. Tobacco smoking, cooking, kerosene heating [15], gas burners [15,16], burning of candles [17], incense sticks [18] and biomass in open fireplaces [8,19,20,21] are the main indoor sources of fine and ultrafine particles [22,23,24,25]. In recent studies, a considerable attention was paid to indoor biomass combustion [26] that releases a considerable amount of pollutants, including carbon monoxide (CO), nitric oxides (NO*_x_*), sulfur dioxide (SO_2_), formaldehyde (HCHO), volatile organic compounds (VOC), particulate matter (PM) and polycyclic aromatic hydrocarbons (PAHs) [27,28,29]. Ventilation systems and other heat sources can influence indoor air quality determining higher pollutant levels than outdoors [30,31,32,33]. Moreover it was found that combustion processes contribute poorly to 24-h mean PM_10_ levels [8,34]. However, number concentrations of emitted fine particles (PM_2.5_) and UFP (particles with diameter less than 100 nm) are relevant and thus, may be a more appropriate predictor of health effects [35,36]. Therefore, several authors have paid particular attention to the formation of PM and UFP in indoor air when operating wood-burning fireplace ovens and stoves [36,37,38,39,40,41]. These studies proved that these heating systems were potential sources of particles [42]. In detail, the number concentration and chemical composition of the particles emitted by open fireplace and stoves are key elements for indoor exposure assessment and for developing appropriate mitigation strategies. Therefore, this work aimed to evaluate the impact of wood fireplaces and stoves on indoor pollutant concentrations and to study the dynamics of pollution phenomena. In particular, 12-h PM_10_ samples were collected in six residential houses located in the hinterland of Bari (Southern Italy) in order to measure their mass and PAH concentrations for evaluating the residents’ exposure. Simultaneously, real time monitoring of PM_10_, PAHs, UFP and Total Volatile Organic Compounds (TVOC) was carried out in order to study the progress of pollution phenomena due to biomass burning.

## 2. Experimental Section

Indoor air quality was measured in six houses in the Apulia Region characterized by a heating system based on wood burning stoves or fireplaces. Houses 1 and 3 have cast iron wood stoves while the other houses have open fireplaces as heating system, respectively. The house and heating system characteristics and information regarding type and weight of wood are reported in Table 1.

**Table 1:** Type of heating systems used, amount and type of wood burned and room volume for each house.

Monitored Houses	Heating System	Amount of Wood Burned (kg)	Type of Wood Burned	Room Volume (m^3^)
House 1	Wood stove	16	Olive tree wood	98.0
House 2	Fireplace	15	Olive and almond tree wood	72.0
House 3	Wood stove	20	Olive and pine wood	42.0
House 4	Fireplace	18	Olive and almond tree wood	40.0
House 5	Fireplace	12	Olive tree wood	103.0
House 6	Fireplace	18	Olive tree wood	56.0

Monitoring campaign of 48-h periods was performed in each house for assessing indoor PM_10_ concentrations and particle size distribution. For the duration of the monitored periods, two 12-h PM_10_ samples were collected in each house during biomass burning and two 12-h PM_10_ samples during no-burning periods. In particular, PM_10_ was collected by a Sequential Air Sampler (SILENT Sequential Air Sampler—FAI Instruments S.r.l., Roma, Italy) for 12 h on polycarbonate fiber filters (47 mm diameter Whatman, Buckinghamshire, UK) equipped with sampling heads operating at a flow rate of 10 L/min with a relative uncertainty of 5% of the measured value. A total amount of 24 PM_10_ samples were collected and stored in a freezer at −4 °C. PM_10_ samples were analyzed for determining PAH concentrations. The extraction of PAHs was conducted with a mixture of acetone/hexane through a microwave assisted solvent extraction by a Milestone, model Ethos D device (Milestone s.r.l., Sorisole (BG) Italy), which allowed the simultaneous extraction of up to 10 samples under the same conditions. The extracted samples were analyzed using an Agilent 6890 PLUS gas chromatograph (Agilent Technologies, Inc., Santa Clara, CA, USA) equipped with a programmable temperature vaporization injection system (PTV) and interfaced with a quadrupole mass spectrometer, operating in electron impact ionization (Agilent MS-5973 N, Inc., Santa Clara, CA, USA. The identification of each PAH (benzo[a]anthracene (BaA), benzo[b+j]fluorene (Bb+jF), benzo[k]fluoranthene (BkF), benzo[a]pyrene (BaP), benzo[g]perylene (BgP), indenopyrene (IP) and dibenzoanthracene (DBA)) was performed using perylene D_12_ (PrD, 264) as the internal standard (IS). The analytical performance of the whole procedure (extraction recovery, extraction linearity, analytical repeatability, Limit Of Detection-LOD) was verified in our previous study [43].

**Figure 1:**
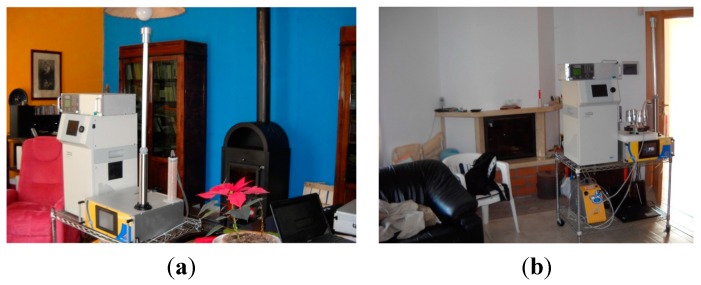
Instrument positioning in (**a**) House 1 and (**b**) House 5.

Moreover, the high time resolved concentrations of PM_10_ and ultrafine particles were measured. The real time PM_10_ concentration was provided by an Optical Particle Counter (OPC Multichannel Monitor—FAI Instruments, Roma, Italy). Number concentration and size distribution of ultrafine particles ranged from 5.6 nm to 560 nm were measured by using a Fast Mobility Particle Sizer (FMPS 3091—TSI, Buckinghamshire, UK. Simultaneously the real time concentrations of total VOC and total PAHs were monitored by a PID PhoCheck TIGER (Ion Science Inc., Cambridge, UK) and by an Ecochem PAS 2000 (SARAS S.p.A., S.p.A., Milno, Italy), respectively. The instruments were installed in front of the heating system, at a height of 1.5 ± 0.1 m from the ground and 1.0–1.5 m away from any door or vent. All sampler inlets were placed at least 1 m from any other combustion source (Figure 1).

A questionnaire was administered to habitants in order to obtained information about stove or fireplace use, the amount of wood used during the sampling period, ventilation conditions and the occupants’ normal behavior (cooking and heating appliances, hygiene and personal care, use of sanitation and cleaning products, *etc.*).

## 3. Results and Discussion

The average PM_10_ and PAH concentrations obtained during the biomass burning periods (two 12-h PM_10_ filters) and no-burning periods (two 12-h PM_10_ filters) are showed in Table 2.

**Table 2:** PM_10_ and PAH concentrations obtained during biomass burning (lighting) and no-burning (no lighting) periods in house operating wood stove (Houses 1 and 3) and fireplace (Houses 2, 4–6).

Monitored Houses	Activities	B(a)A	B(b+j)F	B(k)F	B(a)P	DBA	IP	BgP	∑PAH (ng/m^3^)	PM_10_ (ng/m^3^)
House 1	Lighting	73.8	31.1	9.7	19.7	20.9	19.5	38.0	212.7	66.3
	No lighting	29.5	26.6	8.7	16.6	15.1	15.8	27.6	139.9	54.2
House 2	Lighting	97.0	22.5	9.1	12.9	11.0	10.6	22.8	185.9	74.8
	No lighting	34.3	12.1	3.9	6.5	8.7	7.4	16.1	89.0	54.7
House 3	Lighting	46.6	12.0	4.0	4.1	7.3	5.9	13.0	92.9	212.3
	No lighting	4.7	5.1	2.6	3.2	3.3	3.1	5.5	27.5	52.9
House 4	Lighting	99.5	17.7	5.4	16.6	12.8	9.3	21.5	182.8	80.7
	No lighting	24.6	14.7	5.1	9.3	9.9	8.6	20.0	92.3	53.8
House 5	Lighting	52.1	19.7	14.1	11.9	14.2	9.4	24.3	145.7	38.2
	No lighting	30.3	11.1	4.0	5.0	14.7	7.5	17.3	89.9	22.8
House 6	Lighting	45.5	11.6	3.2	5.6	8.6	6.1	12.5	93.0	67.1
	No lighting	1.7	2.6	1.3	1.5	2.0	1.7	2.9	13.7	45.9

The results obtained during the monitored periods showed that the highest concentrations of PM_10_ were detected when operating the wood burning fireplace or stove, highlighting the impact of biomass burning on indoor air quality. PM_10_ concentrations were higher than the limit value (50 μg/m^3^) established by the Directive 2008/50 European Commission for outdoor environments, except for House 5. The highest concentrations were determined in House 3 (212.3 μg/m^3^ and 52.9 μg/m^3^ with the fireplace on and off, respectively) where a cast iron closed wood stove was placed in an environment with lower ceilings. On the contrary, the lowest concentrations of House 5 (38.2 μg/m^3^ and 22.8 μg/m^3^ during biomass burning and no-burning, respectively) could be due to the larger and better ventilated monitored environment. The same findings were observed for PAHs. The sum of PAH concentrations ranged from 92.9 to 212.7 and from 13.7 to 139.9 ng/m^3^ during the two monitored periods. The highest values were determined in House 1 where a cast iron wood stove was used for domestic heating. BaP concentrations were always higher than the target yearly value of 1 ng/m^3^ established by Directive 2008/50 European Commission for outdoor air, reaching a maximum value of 19.7 ng/m^3^ in House 1 during lighting periods. Moreover, high concentrations of PAHs were detected in the House 5, characterized by the lowest levels of PM_10_. Therefore, this finding suggested that PM_10_ concentration is not a good indicator of indoor air quality.

**Figure 2:**
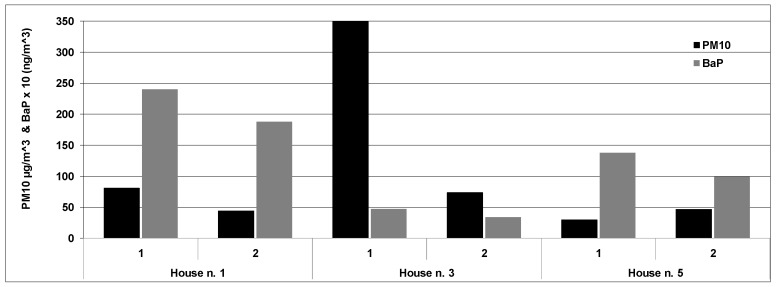
PM_10_ and BaP concentrations for each lighting in three houses.

The comparison between subsequent lightings performed in the same indoor environment showed that many factors could influence the emission process. In detail, it was found that indoor concentrations of PM_10_ and BaP during the two burning periods were different although operating under the same conditions: same heating system, wood burned and indoor environment (see Figure 2).

This finding highlighted that the efficiency of the burning process (combustion duration and temperature, and smoldering or flaming combustion), greatly influence the emissions from fireplaces or wood stoves [11,38].

PAH diagnostic ratios have recently been used as tools for identifying and assessing pollution emission sources [44]. Huang *et al.* found BaP/BgP and IP/[IP+BgP] ratios equal to 1.4–2.0 and 0.64, respectively, during rice straw burning [44,45]. Hays *et al.* showed that BaP/BgP and BaP/IP ratios are strongly linked to wheat residue burning in an experimental chamber [46]. In our previous study of sources conducted in olive tree fields, the BaP/BgP, IP/[IP+BgP], BaP/IP and IP/BgP ratios were found [47]. These average ratio values for olive wood burning were compared with those of this study during biomass burning and no-burning periods (Table 3). Good agreement between the diagnostic ratios obtained from olive wood combustion and those in indoor environments was not found, suggesting that several sources in indoor environments contribute to PAH concentrations influencing the diagnostic ratio.

**Table 3:** The average of diagnostic ratios comparison between this study (lighting and no lighting) and biomass burning in olive trees field.

Diagnostic Ratios	Biomass Burning in Olive Tree Fields [47]	This Study	
		Lighting	No Lighting
IP/BgP	1.12	0.5	0.5
IP/(IP+BgP)	0.53	0.3	0.3
BgP/BgP	1.55	0.5	0.5
BgP/IP	1.38	1.1	0.9

Moreover, no significant differences were observed between the diagnostic ratios associated with biomass burning and no-burning periods, both for fireplaces and wood stoves. This result suggested that the impact of biomass burning source on IAQ was also relevant when the fireplace and stove were not operated.

**Figure 3:**
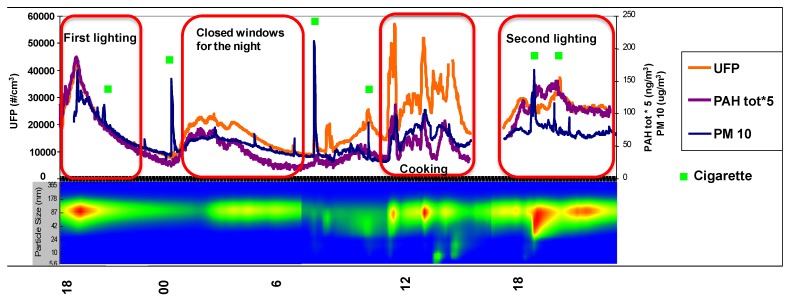
Temporal trend of PM_10_, UFP and total PAH concentrations in House 1. The FMPS spectrum is reported in the bottom picture.

Therefore, the real time concentrations of PM_10_, UFP, total PAHs and VOCs were monitored in each house. The VOC concentration results are not included in the paper because no relevant information concerning biomass burning as an indoor VOC source were obtained. On the contrary, the high time resolved data of PM_10_, UFP and total PAH concentrations, highlighted the impact of several indoor sources such as lighting, cigarettes and cooking. As an example, Figure 3 shows the temporal trend of these pollutants in House 1. The two biomass burning periods showed different trends of investigated pollutants in terms of intensity and duration, confirming the above. Real time monitoring of pollutants also showed that the habitants’ behavior may have a great influence on indoor pollutant emissions and thus on indoor air quality. In fact, it was found that closing the windows during the night after the biomass burning determined a pollutant increase due to their stagnation in the indoor environment. This finding suggests that ventilation frequency and duration, fireplace characteristics, design and location are the key elements for improving IAQ. In detail, it was found that PAH, PM_10_ and UFP concentrations were lower in large and well-ventilated environments. In particular, in House 5 the fireplace was characterized by a strong chimney draft and it was positioned in a big room, an open space where there was access to three other rooms and a stairway leading upstairs.

In addition, UFP concentrations determined in all houses provided the same results concerning BaP: the highest concentrations were determined in House 1 (daily mean: 2.2 × 10^4^ particles/cm^3^), while the lowest one in House 3 (daily mean: 1.4 × 10^4^ particles/cm^3^). Moreover, the UFP concentrations during biomass burning in fireplaces ranged from 1.8 × 10^4^ to 4.5 × 10^4^ particles/cm^3^ and the highest concentrations were detected during food cooking, while sharp peaks were observed during cigarette smoking. In particular, the mean UFP concentration during food cooking was 4.8 × 10^4^ particles/cm^3^ and it reached maximum values of 5.8 × 10^4^ particles/cm^3^. The number of UFP emitted during cigarette smoking was variable and reached a maximum value of 5.2 × 10^4^ particles/cm^3^. The FMPS spectrum also revealed the nucleation and accumulation period of UFP. In particular, the first lighting resulted in midday “blobs” in the spectrum suggesting nucleation bursts of particles around 80 nm. On the contrary, the second lighting was characterized by a nucleation event with “banana-like” growth characteristics, indicating that coagulation occurred when emitted particles reached high concentrations.

In fact, as reported in previous works, coagulation occurs when Brownian motion determines collisions with surrounding gas molecules producing a shift in UFP size [48,49,50]. The differences between the two lightings could be due to the better dispersion conditions during the first event that do not allow particle agglomeration and growth. The other two nucleation events with the “banana-like” growth characteristics were determined in the hours when coffee and lunch were cooked.

**Figure 4:**
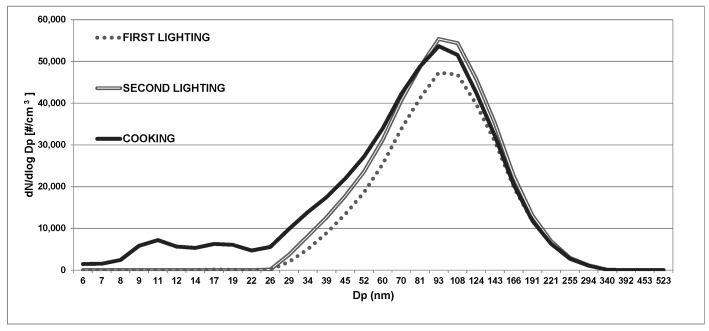
Size distribution of UFP emitted during the two lightings and cooking for House 1 (for example).

The size distribution of UFP emitted by biomass burning in fireplaces was unimodal with a primary mode from 70 to 90 nm. The same trend was determined for the UFP distributions during cooking (in Figure 4), when the UFP concentrations reached 5.8 × 10^4^ particles/cm^3^. However, even if the particles in accumulation mode were comparable between biomass burning and cooking sources, higher concentrations of UFP with diameters ranging from 10 to 20 nm were registered for cooking sources. This result is in agreement with previous studies where frying produced peak number concentrations of UFPs at about 70 nm, with a secondary peak at 10 nm [51,52,53]. These results confirmed the high impact of cooking on indoor air quality.

## 4. Conclusions

The present study enables us to highlight the impact of biomass burning on indoor air quality. In fact, an increase of PM_10_ and BaP concentrations were detected when operating open wood burning fireplaces and stove, reaching values higher than the limit and target values set by Directive 2008/50 European Commission. The 12 h PM_10_ sampling and PAHs diagnostic ratio highlighted the relevant impact of biomass burning source on IAQ, even if fireplaces and stoves were not operated. The real time monitoring was a useful tool: (a) to study the dynamics of pollution phenomena due to biomass burning; (b) to identify the different indoor sources and (c) to evaluate the factors influencing the indoor pollutant concentrations. The study showed that ventilation frequency and duration, fireplace characteristics, design and location could be the key elements for improving the indoor air quality and preserving human health.
